# Transporters involved in pH and K^+^ homeostasis affect pollen wall formation, male fertility, and embryo development

**DOI:** 10.1093/jxb/erw483

**Published:** 2017-02-23

**Authors:** Senthilkumar Padmanaban, Daniel D Czerny, Kara A Levin, Alexander R Leydon, Robert T Su, Timothy K Maugel, Yanjiao Zou, Salil Chanroj, Alice Y Cheung, Mark A Johnson, Heven Sze

**Affiliations:** 1 Department of Cell Biology & Molecular Genetics, University of Maryland, College Park, MD, USA; 2 Department of Molecular Biology, Cell Biology, and Biochemistry, Brown University, Providence, RI, USA; 3 Laboratory for Biological Ultrastructure, University of Maryland, College Park, MD, USA; 4 Department of Biochemistry and Molecular Biology, University of Massachusetts, Amherst, MA, USA; 5 Department of Biotechnology, Burapha University, Chon-Buri, Thailand; 6 Department of Plant Science and Landscape Architecture, University of Maryland, College Park, MD, USA; 7 Maryland Agricultural Experiment Station, University of Maryland, College Park, MD, USA

**Keywords:** Cation, endomembrane, male fertility, pollen, proton, transporter, wall formation

## Abstract

Flowering plant genomes encode multiple cation/H^+^ exchangers (CHXs) whose functions are largely unknown. AtCHX17, AtCHX18, and AtCHX19 are membrane transporters that modulate K^+^ and pH homeostasis and are localized in the dynamic endomembrane system. Loss of function reduced seed set, but the particular phase(s) of reproduction affected was not determined. Pollen tube growth and ovule targeting of *chx17chx18chx19* mutant pollen appeared normal, but reciprocal cross experiments indicate a largely male defect. Although triple mutant pollen tubes reach ovules of a wild-type pistil and a synergid cell degenerated, half of those ovules were unfertilized or showed fertilization of the egg or central cell, but not both female gametes. Fertility could be partially compromised by impaired pollen tube and/or sperm function as *CHX19* and *CHX18* are expressed in the pollen tube and sperm cell, respectively. When fertilization was successful in self-pollinated mutants, early embryo formation was retarded compared with embryos from wild-type ovules receiving mutant pollen. Thus CHX17 and CHX18 proteins may promote embryo development possibly through the endosperm where these genes are expressed. The reticulate pattern of the pollen wall was disorganized in triple mutants, indicating perturbation of wall formation during male gametophyte development. As pH and cation homeostasis mediated by AtCHX17 affect membrane trafficking and cargo delivery, these results suggest that male fertility, sperm function, and embryo development are dependent on proper cargo sorting and secretion that remodel cell walls, plasma membranes, and extracellular factors.

## Teaser

Endomembrane system cation/H^+^ exchangers affect male gametophyte and sperm function through membrane trafficking that is likely to remodel the plasma membrane and cell walls.

## Introduction

Early land plants, such as mosses and ferns, reproduce using motile male gametes that can swim through an aqueous medium to the female gametes. However, in flowering plants, two sperm cells are carried by a male gametophyte (pollen) to the egg cell and the central cell in the absence of an aqueous environment. After a pollen grain lands on a receptive stigma, it germinates and extends a pollen tube inside pistil tissues that surround the female gametophyte. Once inside the embryo sac, the tube ruptures to release two sperm. One fuses with the egg cell and the other with the central cell to complete double fertilization (Dresselhaus and Franklin-Tong, 2013). Subsequent development of the embryo and the endosperm produces a desiccated seed that can remain viable for long periods of time. Although the major steps of reproduction have been known for decades, the molecular and cellular bases of pollen–pistil interactions that culminate in successful double fertilization are not well understood.

Recent studies have identified key molecular players in pollen–embryo sac interactions, including pollen tube attractants secreted by synergid (Okuda *et al.*, 2009; Higashiyama and Takeuchi, 2015) and egg cells (Márton *et al.*, 2005); receptor-like kinases at the plasma membrane (PM) of pollen (Boisson-Dernier *et al.*, 2009) and synergid cells (Escobar-Restrepo *et al.*, 2007); and cell surface proteins of the sperm (HAP2/GCS1) (Mori *et al.*, 2006; von Besser *et al.*, 2006). Pollen germination and tip growth are accompanied by fluxes and oscillations of [Ca^2+^], [K^+^], or pH (Feijó *et al.*, 2001). Many transporter genes are preferentially expressed in pollen grains and tubes (Bock *et al.*, 2006; Qin *et al.*, 2009), though their biological roles are mostly unknown. ACA9 is a pollen-specific Ca^2+^ extrusion pump that is localized to the pollen tube PM. T-DNA insertional mutants show reduced pollen tube growth in vivo and aborted fertilization due in part to failed pollen tube rupture (Schiott *et al.*, 2004). Thus disruption of Ca^2+^ efflux by ACA9 probably caused a defect in Ca^2+^ signaling, resulting in slowed tube growth, failed tube rupture, and reduced seed set. A single mutant of a cyclic nucleotide-gated ion channel, *cngc18*, showed male sterility due to a defect in pollen tube growth and inability to enter the transmitting tract (Frietsch *et al.*, 2007). However, the single mutants *cngc7* or *cngc8* showed no defect, though pollen grains of the double mutants were sterile, possibly due to a tendency to burst before the tube emerged (Tunc-Ozdemir *et al.*, 2013). These experiments suggest that cNMP may stimulate Ca^2+^ influx or oscillations required for pollen germination and tip growth. Very little is known about transporters that mediate pH and cation homeostasis in pollen–female gametophyte interactions.

The diversification of one transporter subfamily, CHXs (cation /H^+^ exchangers), in monocot and eudicot plants relative to early land plants is striking, especially as ~18 *Arabidopsis thaliana* CHX genes are expressed in pollen (Sze *et al.*, 2004). The Cation/Proton Antiporter1 (CPA1) subfamily includes mammalian NHE and plant NHXs (Brett *et al.*, 2005; Chanroj *et al.*, 2012). The CPA2 subfamily (including AtCHX) is predominantly conserved in bacteria, archea, fungi, amoebozoa, and plants, but not in metazoa (Chanroj *et al.*, 2012). Basal land plants, such as moss, contain 3–4 copies of *CHX* genes per genome (Mottaleb *et al.*, 2013), but ~17 and 28 members are predicted in rice and Arabidopsis, respectively. Their biological roles are mostly unexplored. We proposed that the diversification of CHXs in flowering plants is related to reproductive success (Chanroj *et al.*, 2012), and the ability to survive on land.

Several studies support the idea that expansion of the *CHX* gene family facilitated colonization of land. First, *CHX21* and *CHX23* were necessary for pollen tube targeting to ovules in *A. thaliana*. Mutant *chx21chx23* pollen grains germinate, but tubes fail to turn towards the ovule and thus do not reach the micropyle. Thus, *CHX21* and *CHX23* could function either in pollen tube perception of female guidance cues or in signal transduction to mediate a change in growth direction (Lu *et al.*, 2011). Secondly, *AtCHX20* in guard cells facilitates light-induced stomatal opening (Padmanaban *et al.*, 2007). Localized to reticulate membranes resembling the endoplasmic reticulum (ER), CHX20 could regulate osmoregulation via membrane trafficking needed to increase vacuolar and PM area. Recently, *GmSALT3* or *GmCHX1*, which encodes a homolog of AtCHX20, was shown to confer salt tolerance in certain soybean varieties (Guan *et al.*, 2014; Qi *et al.*, 2014).

The best characterized CHXs from *A. thaliana* show roles in pH and cation homeostasis. *CHX17*, *CHX18*, or *CHX19* are localized to the pre-vacuolar compartments (PVCs) and the PM in plant cells (Chanroj *et al.*, 2011, 2013). Their expression in an alkaline-sensitive yeast strain conferred tolerance to growth at pH 7.5, suggesting a role in pH homeostasis. CHX17 also mediated K^+^ transport, as its expression in *Escherichia coli* strains deficient in K^+^ uptake pathways restored growth on low K^+^ medium and mediated ^86^Rb^+^ uptake. Furthermore, *CHX17* as well as *CHX18* and *CHX19* restored growth of yeast lacking K^+^ uptake transporters (Chanroj *et al.*, 2011). These results in yeast are consistent with their role as a K^+^/H^+^ antiporter at the endomembrane; though the mode of transport at the PM is less clear (Chanroj *et al.*, 2011; Mottaleb *et al.*, 2013). Structural modeling predicts that AtCHX17 protein has an NhaA-fold architecture, and mutagenesis showed core residues at positions similar to cation/H^+^ exchangers (Czerny *et al.*, 2016).

The roles of *CHX17*, *CHX18*, and *CHX19 in planta* are less clear. *CHX17* is expressed in roots, though vegetative growth of seedlings in single, double, or triple mutants (*chx17chx18chx19*) under various stress conditions was unaltered (Chanroj, 2011). Curiously, quadruple mutants (*chx16chx17chx18chx19*) were recovered at a lower frequency than expected. Furthermore, ther quadruple mutant pod contained 60% fewer seeds than the wild type (Chanroj *et al.*, 2013). Homozygous triple mutants, *chx17chx18chx19*, were also recovered at a lower than expected frequency. Here, we determine which phase of reproduction led to reduced seed set in mutants. We demonstrate that *chx17chx18chx19* pollen tubes grow, target, and enter ovules; however, many targeted ovules fail to develop into seeds, suggesting a failure to complete fertilization. Our findings underscore a previously unknown role for endomembrane transporters and K^+^ and pH homeostasis in male fertility, fertilization, and embryo development possibly through remodeling of the cell wall and PM.

## Materials and methods

### Plants and genotyping

*Arabidopsis thaliana*, Columbia-0, was grown in Miracle-Gro^®^ potting mix supplemented with 5% Perlite. Plants were grown under a 16 h photoperiod at 150 μE m^–2^ s^–1^ illumination, 21 °C, and 60% relative humidity. All *chx* mutants used (see Supplementary Table S1 at *JXB* online) are available from ABRC.

Genotype was determined by PCR using two gene-specific primers or one gene-specific primer and a T-DNA border primer (Supplementary Table S2). Genomic DNA was isolated from 1–2 rosette leaves of ~5-week-old plants (Edwards *et al.*, 1991). DNA was amplified by *Taq* DNA polymerase (NEB M0273L) in a three-step PCR program for 40 cycles. PCR products were separated on a 1% agarose gel and visualized using ethidium bromide.

### Segregation analysis

To test male transmission of the mutant allele, pollen from a parent carrying a heterozygous gene was transferred to a pistil carrying the wild-type gene. To test female transmission, a stigma heterozygous for a gene was given pollen harboring the wild-type gene. Parental plants were genotyped for wild-type genomic sequence or T-DNA insertions in *CHX17*, *CHX18*, and *CHX19* genes. Stage 12 flower buds (Smyth *et al.*, 1990) were emasculated, and pistils were pollinated 2 d later, with anther from stage 13 flowers. Seeds developed on the plant for 10–12 d until pods dried. F_1_ seeds were planted, and genomic DNA from ~100 plants was extracted for genotype analyses.

### *Analysis of wild-type and* chx17/18/19 *plants*

Siliques from the primary bolts of 9-week-old plants were collected starting from the fourth pod below the inflorescence. Pod lengths were measured in ImageJ (NIH) using at least 10 plants per line. Four pods of average length per plant were split open and seeds were scored under a stereomicroscope (Nikon SMZ1000).

To detect nuclei, pollen grains from 1–3 stage 13 flowers (Smyth *et al.*, 1990) were dabbed onto a microscope slide, and incubated with 30 µl of DAPI solution for 15 min. DAPI (Life Technologies D3571) solution contained 0.1 M sodium phosphate pH 7, 1 mM EDTA, 0.1% Triton X-100, and 0.4 µg ml^–^1 DAPI. Pollen was examined by Nikon E600 with a UV filter (excitation/emission at 360/410 nm).

To visualize pollen tubes *in vivo*, pollinated pistils were stained with aniline blue to label callose (Mori *et al.*, 2006). Self-fertilized wild-type and *chx17/18/19* pistils were analyzed 1–4 days after pollination (DAP) (Smyth *et al.*, 1990). Pistils were fixed in 75% ethanol/25% acetic acid for 2 h, and then rehydrated in 75, 50, and 30% ethanol, and deionized water. The pistils were incubated in 8 M NaOH overnight, washed once with deionized water, and then incubated for 2 h in 0.1% decolorized aniline blue (Fisher Scientific A-967) in 100 mM K_2_HPO_4_ at pH 10. Each pistil was placed on a glass slide and pressure was applied to expose ovules and the transmitting tract. Images were recorded using a Nikon E600 fluorescence microscope with a UV filter (excitation/emission wavelengths at 360 nm/410 nm).

Ovule development from self- or manually pollinated pistils was visualized using chloral hydrate. Seed pods were fixed in 90% ethanol/10% acetic acid overnight at room temperature. Pods were washed twice for 30 min in 90% ethanol and then cleared in chloral hydrate/glycerol/water (8:1:2) solution (Berleth and Jurgens, 1993). Each pod was opened and all ovules or developing seeds were mounted in chloral hydrate solution and examined with DIC (differential interference contrast) microscopy.

To examine pollen wall architecture, pollen grains from stage 13 flowers were mounted on stubs over double-sided tape. The specimens were then sputter coated with gold–palladium (60%:40%) (Balzers Med 010) and observed using a scanning electron microscope (SU3500, Hitachi) at an accelerating voltage of 3 kV in high vacuum.

### Monitoring synergid degeneration and gamete fusion

To visualize sperm nuclei, *HTR10:HTR10:RFP* was introduced into the *chx17chx18chx19* mutant by genetic crossing. Self-fertilized *chx* triple mutants with red fluorescent protein (RFP)-labeled sperm were recovered. Oddly, plants showed a range of RFP-labeled pollen, 20–50%, and a few plants with >55% RFP. A triple *chx* mutant containing 73% RFP-labeled pollen was selfed, and progeny yielded flowers in which 85% of the pollen was RFP positive.

Stage 12b or 12c buds (Smyth *et al.*, 1990) were emasculated and hand pollinated 24 h later. Hand pollinations were performed under a dissecting microscope (Zeiss Stemi 2000C) by pollinating emasculated *ACT11:MSI1:GFP* stigmas with pollen from male donor plants carrying *HTR10:HTR10:RFP* (Ingouff *et al.*, 2007). Pollinated pistils were analyzed 16 h after manual pollinations. Pistils were harvested and ovary walls were removed as previously described (Johnson and Kost, 2010). Dissected pistil tissues were mounted in 80 mM sorbitol for analysis by confocal laser scanning microscopy (CLSM) using a ×40 objective with DIC capability (Zeiss LSM800 upright microscope). Green fluorescent protein (GFP) expression was imaged using a diode laser at 10 mW with excitation at 488 nm and emission at 509 nm. mRFP (modified RFP) expression was imaged with a diode laser at 10 mW with excitation at 633 nm and emission at 607 nm. Signal intensities were optimized for each fluorophore and then combined in overlay. Final images represent a merge of single planes at varying depths (*z* stacks). Synergid status was determined based upon the visualization of nuclear *ACT11:MSI1:GFP* signal as described before (Leydon *et al.*, 2015).

### Accession numbers

CHX17 (At4g23700), CHX18 (At5g41610), and CHX19 (At3g17630)

## Results

### chx17chx18chx19 *mutants show reduced seed set*

**Fig. 1.**
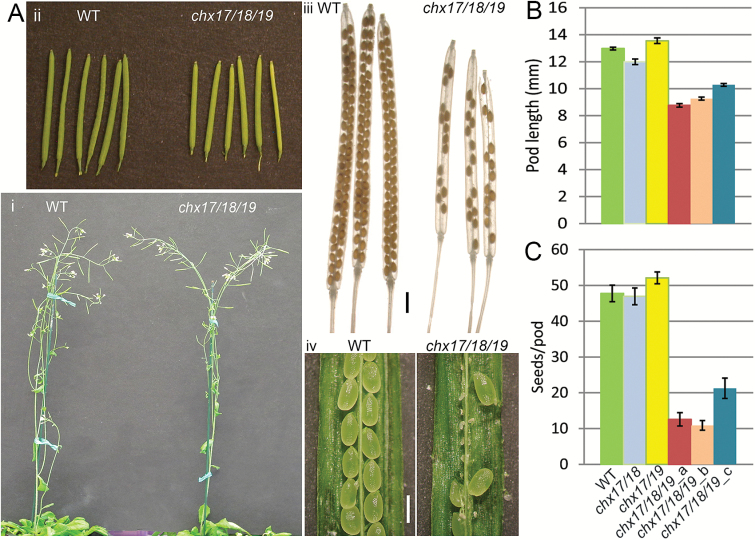
Triple *chx17chx18chx19* mutant plants showed reduced seed set. (A) Plants and seed pods. (i) Wild-type (WT) Columbia-0 and *chx17/18/19* plants have similar vegetative and reproductive growth. (ii) *chx17/18/19* mutant pods were shorter than those of the WT. (iii) *chx17/18/19* pods contained fewer seeds. Scale bar=1.0 mm. (iv) *chx17/18/19* pods contained aborted and developing seeds. At least 44 pods per genotype were analyzed. Scale bar=0.5 mm. (B) Pod lengths of the *chx17/18/19* mutant are 21–29% shorter than pods of the WT or *chx17/18* or *chx17/19* double mutants. Results show the mean, and bars represent the SE (*n*=234–610 pods). (C) Seed number per pod was reduced 55–73% in the *chx17/18/19* mutant relative to the WT or the *chx17/18* and *chx17/19* double mutants. Four siliques of average length were split open per plant to count seeds. Results show the mean, and bars represent the SE (*n*=44–126 pods).

Vegetative growth and flower development of *chx17chx18chx19* plants were similar to those of the wild type (Fig. 1A-i; Supplementary Fig. S1). However, seed pods of *chx17chx18chx19* plants were shorter, varying from 8.8 mm to 10.3 mm, compared with 13.0 mm in the wild type (Fig. 1A-ii, B; Supplementary Fig. S2). When siliques were cleared, mutant pods had random gaps instead of two continuous rows of seeds in wild-type pods (Fig. 1A-iii, iv). Progeny from three *chx17chx18chx19* plants showed, on average, 11–21 seeds, compared with 48 seeds per pod in the wild type (Fig. 1C). Seed set or pod length of double mutants *chx17chx18* or *chx17chx19* were similar to those of the wild type (Fig. 1B, C). Thus, loss of function of three *CHX* genes, *CHX17*,*CHX18*, and*CHX19*, reduced seed set to half that seen in the wild type.

### *Male fertility is compromised in* chx17chx18chx19 *pollen*

**Fig. 2.**
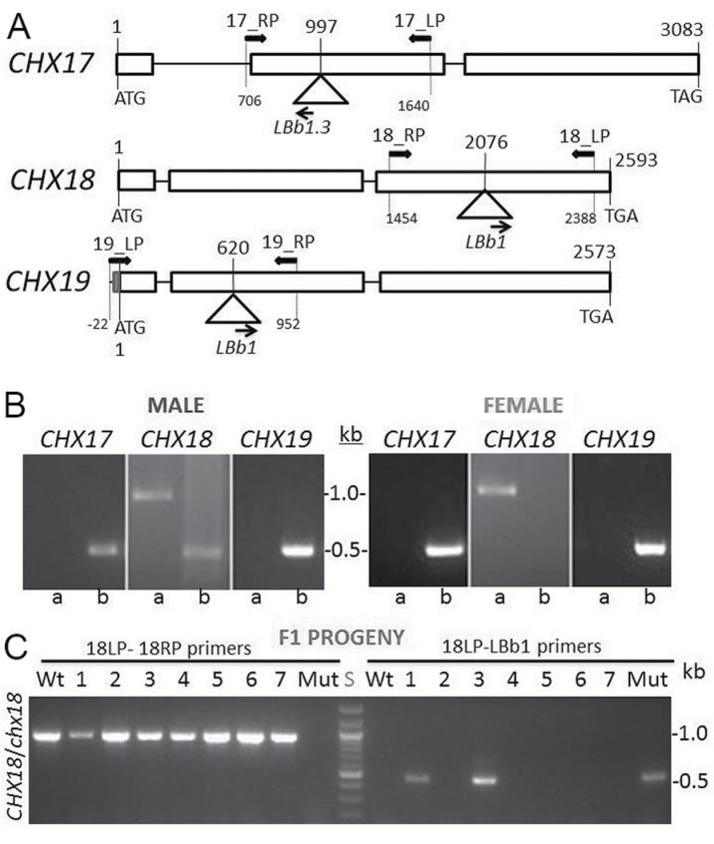
Gene structures of Arabidopsis *CHX17*, *CHX18*, and *CHX19* and verification of genotype. (A) *CHX17*, *CHX18*, and CHX*19* genes and site of T-DNA insertions. Exons and introns are shown as boxes and lines, respectively. Bold arrows indicate right and left border primers (RP and LP) used to amplify the wild-type *CHX* sequence. Arrows refer to the left border primer (LBb1 or LBb1.3) used to detect the T-DNA insertion. Number 1 refers to the first base of the start codon *A*TG, and the number at the end of the gene refers to the third base of the stop codon. Base # above the triangle denotes the position of the T-DNA insertion. *chx17-4*, *chx18-1*, and *chx19-2* correspond to SALK_033417, SALK_001563, and SALK_100047, respectively. Primer sequences are shown in Supplementary Table S2. (B) The genotype of male (*chx17*^*–/–*^*chx18*^*+/–*^*chx19*^*–/–*^) and female (*chx17*^*–/–*^*chx18*^*+/+*^*chx19*^*–/–*^) parents used in a reciprocal cross was verified. Genomic DNA from parents was tested for homozygous or heterozygous *CHX17*, *CHX18*, or *CHX19* genes by PCR. Lane ‘a’ shows fragments amplified with LP and RP primer pairs, and lane b shows fragments amplified using the T-DNA primer with either LP or RP primers. The wild-type allele produces a PCR-amplified product of ~1 kb, whereas mutant alleles give a PCR product of ~0.5 kb. (C) The number of F_1_ progeny heterozygous for CHX18 was less than expected. Lanes 1–7 show a sample of F_1_ plants that were genotyped. Lanes labeled ‘18LP–18RP primer pair’ or ‘18LP–LBb1’ show the PCR-amplified DNA fragment if genomic DNA had a copy of the wild-type *CHX18* or a T-DNA-inserted *chx18*, respectively. Lane ‘S’ is the 100 bp DNA size standard. Lanes WT or ‘Mut’ contained genomic DNA isolated from the wild type or the triple *chx17/18/19* mutant. Plants 1 and 3 are heterozygous for *CHX18*, and plants 2, 4, 5, 6, and 7 are homozygous wild type for *CHX18*.

Segregation distortion in the progeny from a selfed mutant carrying heterozygous CHX18^+/–^ in a double *chx17chx19* mutant background (Chanroj *et al.*, 2013) indicated a gametophytic defect. Reciprocal crosses were conducted to determine whether transmission of T-DNA-disrupted genes was due to the male or the female gametophyte, or both. As double mutants behaved like the wild type, we first tested segregation of a heterozygous *CHX18*^*+/–*^ or *CHX19*^*+/–*^ gene in a double mutant background crossed to the same double mutant. For instance, pollen from a double *chx17*^*–/–*^*chx19*^*–/–*^ mutant plant carrying a heterozygous *CHX18*^*+/–*^ gene was placed onto the same double mutant pistil carrying *CHX18*^*+/+*^ (Table 1). Transmission of *chx18-1* and *chx19-2* mutant alleles was tested by genotyping the F_1_ progeny. Parents were genotyped for mutant or wild-type alleles of *CHX17*, *CHX18*, and *CHX19* by PCR prior to crossing (Fig. 2A, B). The F_1_ progeny were tested for homozygous wild-type *CHX18*^+/+^ or heterozygous *CHX18*^+/–^. PCR-amplified products of 1 kb or 0.5 kb were used to distinguish either the wild-type or mutant allele, respectively (Fig. 2C). If a *CHX* gene has minimal or no effect in male fertility, then progeny containing homozygous wild-type *CHX18*^+/+^ would be approximately equal to progeny carrying heterozygous *CHX18*^+/–^.

**Table 1.** Only one of three *CHX* genes is sufficient to restore male fertility Pollen grains from a double mutant *chx17*^*–/–*^*chx19*^*–/–*^ parent heterozygous for *CHX18*^*+/–*^ were manually transferred to a double mutant pistil carrying wild-type *CHX18*^+/+^ (a). Reciprocal crosses were also conducted (b). Similar reciprocal crosses were performed with the double mutant *chx17*^*–/–*^*chx18*^*–/–*^ heterozygous for *CHX19*^+/–^ (c, d) and the double mutant alone. Seeds from these crosses were planted and leaves were collected for DNA extraction and genotyped. The observed (Obs) segregation of F_1_ progeny is shown below as the number of individuals recovered or percentage (%).

	Female: egg or central cell			Male: pollen or sperm			F _ 1 _ progeny			F _ 1 _ progeny	
	CHX			CHX			CHX			Expect	Obs
	17	18	19	17	18	19	17	18	19	(%)	(%)
a	–	+	–	–	+	–	–/–	+/+	–/–	50	62 (95)
	–	+	–	–	–	–	–/–	+/–	–/–	50	3 (5)
b	–	+	–	–	+	–	–/–	+/+	–/–	50	98 (70)
	–	–	–	–	+	–	–/–	+/–	–/–	50	41 (30)
c	–	–	+	–	–	+	–/–	–/–	+/+	50	92 (97)
	–	–	+	–	–	–	–/–	–/–	+/–	50	3 (3)
d	–	–	+	–	–	+	–/–	–/–	+/+	50	69 (75)
	–	–	–	–	–	+	–/–	–/–	+/–	50	23 (25)

Transmission of the *chx18-1* allele through pollen of the *chx17chx19* double mutant background was 5% instead of the 50% expected (Table 1). Similarly, transmission of the *chx19-2* allele through pollen in a *chx17chx18* mutant background was only 3% instead of 50% (Table 1). In contrast, the average transmission of the *chx18-1* allele through the female was 30% instead of 50% (Table 1). Similarly, transmission of the *chx19-2* allele was 25% instead of 50% (Table 1). Together, these results indicate that *chx17chx18chx19* gametophytes are significantly less fertile than either *chx17CHX18chx19* or *chx17chx18CHX19* double mutants. Thus one functional *CHX* out of three is required for either male or female fertility. We conducted additional reciprocal crosses with the wild type in order to define the impact on male and female reproductive function. When pollen from a double *chx17chx19* mutant that is heterozygous for *CHX18*^+/–^ was transferred to a wild-type pistil, the fertility of the triple *chx* mutant pollen was severely reduced, as only 10% of the F_1_ progeny carried a triple heterozygous genotype (Table 2), a significant deviation from the expected 50%. In the reciprocal cross using wild-type pollen, female transmission of the triple *chx* mutant was 38% instead of 50%, consistent with a role for *CHX* genes in the female gametophyte (Table 2). These data further support our hypothesis that at least one copy of *CHX17*, *CHX18*, or *CHX19* is required for complete male and female gametophyte function. Since the defect in male reproductive function was more pronounced, we focused on defining the roles of *CHX17*, *CHX18*, and *CHX19* in the male gametophyte.

**Table 2.** Reciprocal crosses show that male fertility is compromised in the *chx17chx18chx19* mutant Pollen grains from a double mutant *chx17chx19* parent heterozygous for CHX18 were used to pollinate a wild-type stigma (a). Wild-type pollen grains were used to pollinate a double mutant pistil heterozygous for *CHX18*^+/–^ (b). F_1_ seeds from these crosses were planted and leaves were collected for DNA extraction and genotyped. The observed (Obs) segregation of F_1_ progeny is shown below as number of individuals recovered or percentage (%). Exp. refers to the expected ratio of F_1_ progeny in %.

	Female: egg or central cell			Male: pollen or sperm			F _ 1 _ progeny					
	**CHX**			**CHX**			**CHX**			**Exp**	**Obs**	χ^2^
	**17**	**18**	**19**	**17**	**18**	**19**	**17**	**18**	**19**	**(%)**	**(%)**	
a	+	+	+	–	**+**	–	+/–	+/**+**	+/–	(50)	101 (90)	72.3
	+	+	+	–	**–**	–	+/–	+/**–**	+/–	(50)	11 (10)	
b	–	**+**	–	+	+	+	+/–	+/**+**	+/–	(50)	72 (62)	7.3
	–	**–**	–	+	+	+	+/–	+/**–**	+/–	(50)	43 (38)	

The number of F_1_ plants tested was 112–115.

χ^2^ >6.64 (*P* value of 0.01) indicates that the observed (Obs) results are significantly different from the expected (Exp) Mendelian ratio.

### chx17chx18chx19 mutant *pollen contains three nuclei though the wall pattern is disorganized*

**Fig. 3.**
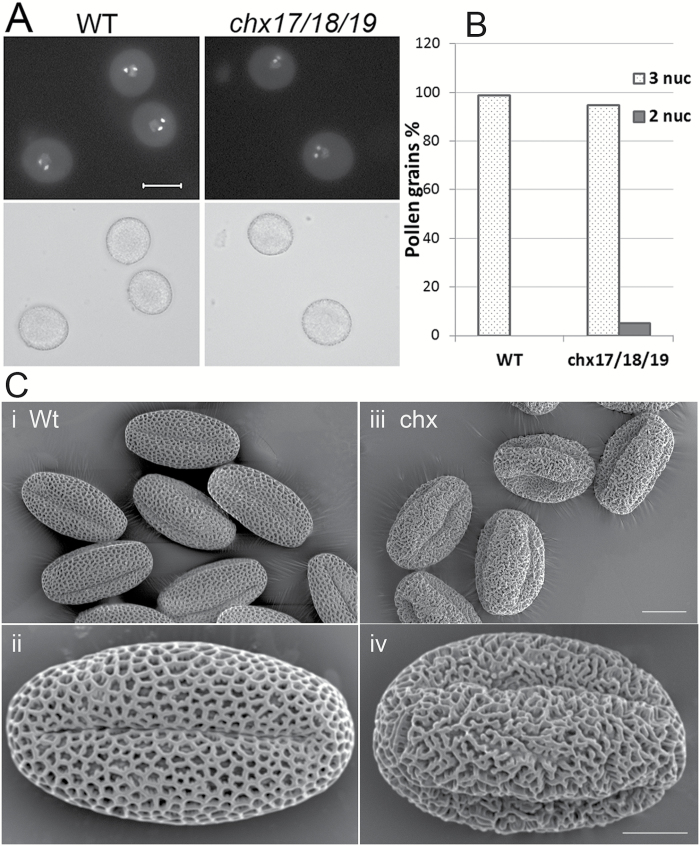
Mutant *chx17chx18chx19* pollen grain contains three nuclei and a disorganized wall pattern. (A) Representative images of DAPI-stained pollen grains observed by UV fluorescence (top) or bright-field (bottom) microscopy. Scale bar=10 µm. (B) Nearly all wild-type and *chx17/18/19* pollen grains contained three nuclei as visualized by DAPI staining. Total grains scored were 108 and 200 from the wild type and *chx17/18/19*, respectively. (C) Loss of reticulate wall pattern. Scanning electron micrographs of the wild type (Wt; i, ii) and *chx17/18/19* mutant pollen grains (iii, iv). Scale bar=10 (i, iii) or 5 μm (ii, iv).

The size and morphology of mutant and wild-type pollen grains from stage 13–14 flowers appeared similar under light microscopy. Pollen grains were stained with DAPI which binds dsDNA. Fluorescence microscopy showed that 95% of triple mutant grains contained three DAPI-stained nuclei like the wild type (Fig. 3A). About 5% of mutant grains showed two DAPI-stained nuclei (Fig. 3B). Thus disruption of *CHX17*, *CHX18*, and *CHX19* genes did not perturb microspore development significantly to produce tricellular pollen.

Intriguingly, SEM showed that the outer wall or exine of all *chx17chx18chx19* mutant pollen grains was disorganized, in contrast to the highly reticulate pattern of wild-type grains (Fig. 3C). The single *chx17* mutant pollen displayed wall sculpture resembling that of wild-type grains (Supplementary Fig. S3). However, the double *chx17chx18* mutant grains gave a mixture of wall patterns where ~52% were fully disorganized, 16% were intermediate, and 31% looked normal (Supplementary Fig. S3). Thus all three *CHX* genes have overlapping roles in pollen wall formation and patterning.

### chx17chx18chx19 *pollen tubes arrive at ovules, but seed development fails*

Pollen tube growth in the pistil was examined using aniline blue. Callose from vascular tissue of the funiculus is distinct as seen in unpollinated pistils. At 1 DAP, wild-type pollen tubes are visible at the base of the pistil (Supplementary Fig. S4). Furthermore, most ovules (74–82%) had received a pollen tube, observed at higher magnification (not shown). The estimate is probably low as many tubes were not visible due to ovule crowding. Two days after wild-type pollination, 93% of seeds (568 out of 611) had enlarged, though pollen tubes were mostly undetectable, suggesting that successful fertilization caused degeneration of pollen tubes. In wild-type pistils pollinated with the triple mutant, many tubes were visible along the length of the pistil at 1 DAP, though fewer tubes were visible at the base, suggesting that pollen germination, tube growth, or both are suppressed. By 2 DAP, some ovules have increased in size, consistent with early seed development after successful fertilization (Supplementary Fig. S2). However a significant number of ovules (≥50%) remained small and are referred to as ‘undeveloped ovules’. At 3 DAP, developing ovules/seed increased in size further; however, the ‘undeveloped ovules’ from triple mutant pollen had begun to shrivel (Supplementary Fig. S4). At 2–3 DAP, we noticed that aniline blue-stained pollen tubes were less or not visible in developing ovules, confirming that the pollen tube degenerated in cases when fertilization is successful.

To distinguish between size differences of undeveloped and developed ovules in pistils pollinated with triple mutant pollen, we analyzed mutants at 2 DAP. We could not see all undeveloped ovules as some are obstructed by the transmitting tract, so we only scored pollen tubes in any undeveloped ovules in full view. For example, we counted 50 pollen tubes inside 132 undeveloped ovules, which included 28–57% pollen reception by undeveloped ovules per pistil. Thus, on average, at least 39% of the undeveloped ovules had received a tube, while a fraction of undeveloped ovules did not receive a tube (Supplementary Fig. S5). In contrast, pistils pollinated with wild-type pollen show that most ovules (86–97%) have increased in size by 2 DAP (Supplementary Fig. S4). Thus mutant pollen grains germinate, extend tubes, and are able to target ovules; however, tube number and tube lengths are reduced relative to the wild type. Even though mutant pollen tube lengths grown *in vitro* for 6 h were 10–20% shorter and the percentage germination was less (14–32%) than in the wild type (58%) (data not shown), on average most (65–80%) ovules received a tube when pollen grains germinated *in vivo* on the stigma.

**Fig. 4.**
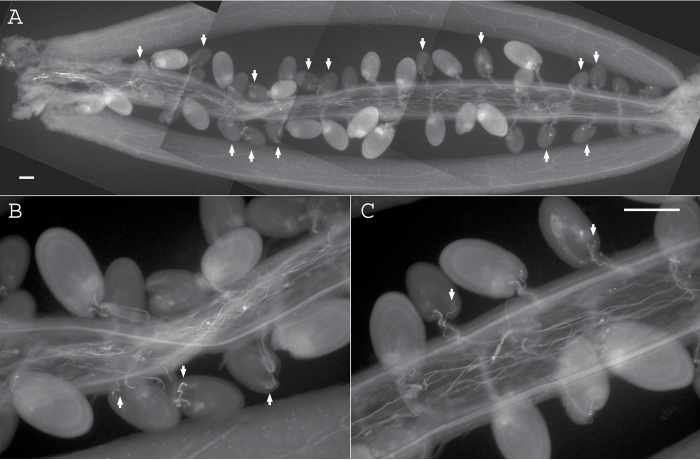
Mutant pollen tubes reached many ovules though half remain undeveloped. Ovules were examined after self-pollination of the triple *chx17chx18chx19* mutant. (A) Some ovules are small and do not develop by 3–4 DAP. Arrows point to visible pollen tubes inside undeveloped ovules. (B, C) Magnified images of the same pod show that aniline blue-stained tubes have entered ovules that remain undeveloped. The vascular strand is visible at the chalazal end of each ovule. Images are representative of 6–10 pods. Scale bar=100 µm. (A) is a composite of frames imaged using a ×10 objective lens and (B) and (C) are imaged with a ×20 objective.

We found that growth of mutant pollen tubes in self-pollinated triple mutants is slightly more robust than that manually transferred to wild-type pistils. In pods of self-pollinated wild-type plants, fluorescent-labeled pollen tubes were visible outside each developing ovule/seed (not shown). Mutant *chx17chx18chx19* pollen tubes also grew through the female transmitting tissues to the distal end of the pod, as evident from developing seeds (Fig. 4A). Though triple mutant tubes did reach the distal end, many small undeveloped ovules were seen along the entire length of the pod. Upon closer examination, a pod at the right developmental stage (2–3 DAP) showed that 15 out of 21 undeveloped ovules had received a tube (Fig. 4A). Some undeveloped ovules at the distal end of the pod were homogeneously gray and did not receive a tube (Fig. 4A, far right) thus they were unfertilized. Other undeveloped ovules with a visible pollen tube showed bright grainy content (Fig. 4C, right), suggesting abnormal development after tube reception. Out of three pods at a similar developmental stage, we counted 41 pollen tubes entering 65 undeveloped ovules, indicating that at least 63% of undeveloped ovules have received a tube. In three separate experiments using 10 pods each, we detected that pollen tubes had entered 30–63% of the undeveloped ovules in the pistils from the triple mutant (Fig. 4B, C). These results indicated that the pollen–pistil interactions regulating *in vivo* pollen tube growth and tube guidance of the triple mutant were not perturbed. Mutant tubes are able to sense chemical cues in the pistil and those from the embryo sac and respond by targeting the micropyle. The results indicate that even though pollen tubes reach ovules, there is a failure to complete fertilization.

### chx17chx18chx19 *pods contain unfertilized ovules and single fertilization events*

**Fig. 5.**
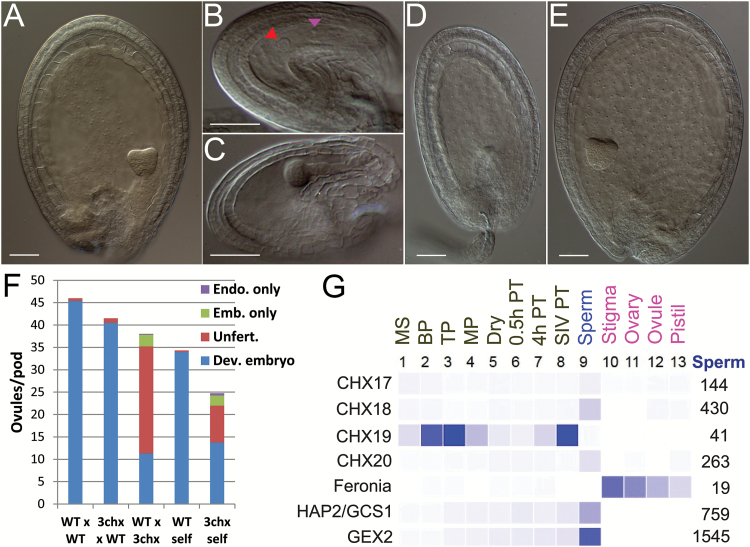
Pistils receiving *chx17chx18chx19* mutant pollen produced unfertilized ovules, embryo only, and developing seeds. (A) Wild type (WT) control of developing seed at ~3 DAP. Pods were cleared with chloral hydrate and examined by microscopy. (B–E) Triple mutant ovules ~3 d after self-pollination. (B) Unfertilized ovule shows an egg (arrowhead) and a central cell (red arrowhead). (C) Globular embryo only. (D) Endosperm only. (E) Heart stage embryo. Scale bar=50 µm. (F) Distribution of ovule development per pod after reciprocal or self-pollination. Manual pollinated pistils: WT pistil receiving WT pollen (WT×WT); *chx17/18/19* mutant (3chx) pistil receiving WT pollen (WT×3chx), and WT pistil receiving *chx17/18/19* mutant pollen (3chx×WT). ‘WT’ self or ‘3chx self’ refers to pods of the self-pollinated WT or c*hx17/18/19* mutant. The mumber of ovules per pod was based on 10–14 pods (manual crosses) or 3–6 pods (self). ‘Unfert.’ indicates unfertilized ovule (as in B); ‘Emb’ or ‘Endo’ refers to embryo (C) and endosperm (D) only, and ‘Dev’ refers to developing embryo (as in E). (G) Expression of Arabidopsis *CHX18* and *CHX19* in sperm and pollen based on the ATH1 transcriptome. Heat map showing normalized transcriptome results (http://arabidopsis-heat-tree.org/) of *CHX* and other characterized genes in microspore (MS), bicellular pollen (BP), tricellular pollen (TP), and mature pollen (MP) (Honys and Twell, 2004), dry pollen grains (Dry), pollen tubes germinated for 0.5 h (0.5 PT), 4 h (4h PT), or in tubes emerging from a cut style after grain germination on a stigma (SIV, semi-*in vivo*) (Qin *et al.*, 2009), and sperm cells (Borges *et al.*, 2008). Gene expression in female tissues, include stigma and ovary (Schmid *et al.*, 2005), ovule and unpollinated pistil (pistil) (Boavida *et al.*, 2011). Normalized values of expression in sperm are shown on the far right. Feronia (At3g51550) is expressed in synergid cells. HAP2/GCS1 (At4g11720) and GEX2 (At5g49150) are controls for sperm-expressed genes.

Pods of manually or self-pollinated flowers were treated with chloral hydrate, and every ovule per pod was examined by DIC microscopy 3–4 DAP. Wild-type pistil pollinated with triple mutant *chx17chx18chx19* pollen yielded three types of ovules: (i) unfertilized ovules (63%); (ii) single-fertilized ovules containing either an embryo only or a small endosperm only (7%); and (iii) normal seeds that contain both developing embryo and endosperm (30%) (Fig. 5F). Pods from the control wild type yielded nearly all normal seeds (98%), similar to pods obtained from triple mutant pistils pollinated by wild-type pollen (98%) (Fig. 5F). These results are consistent with the reciprocal cross study where male fertility is severely compromised by loss of function of three *CHX* genes in the male gametophyte.

Pods of self-pollinated triple *chx17chx18chx19* mutants also contained three classes of ovules: (i) 33% unfertilized ovules; (ii) 11% single-fertilized ovules; and (iii) 55% normal developing seeds (Fig. 5A–E). The percentage of undeveloped ovules that includes unfertilized and single fertilized ovules ranged from 45% to 70% based on analysis of six pods. Unfertilized ovules were verified by the presence of a central nucleus or egg cell, as shown in Fig. 5B. Products of single fertilization, though less frequent, were consistently observed mostly as a globular embryo only (Fig. 5C). We also noticed developing endosperm without any visible embryo (Fig 5D). These results indicate that only one fertilization event was successful. Ovules with endosperm alone varied in size, and can increase to twice the length of the unfertilized ovule (Fig. 5D). About one-third to half of the ovules per pod developed normally, judging by the development of both an embryo and an endosperm, indicating that a subset of triple mutant pollen was competent to complete double fertilization with triple mutant female gametes (Fig. 5E).

### chx17chx18chx19 *pollen tubes arrive at the female gametophyte but show reduced sperm delivery and gamete interaction defects*

**Fig. 6.**
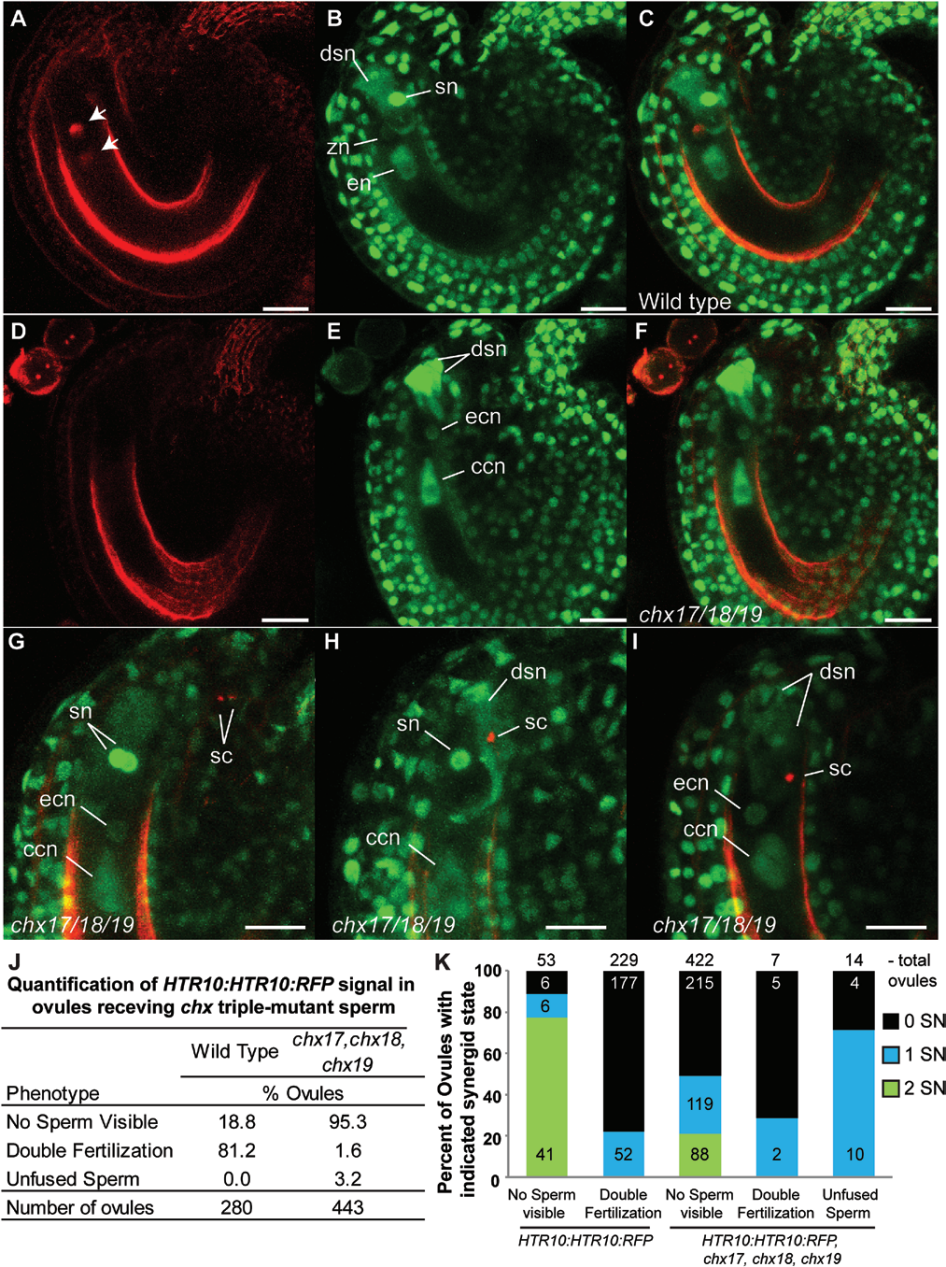
Live imaging demonstrates that *chx17chx18chx19* pollen tubes trigger synergid degeneration, yet sperm frequently fail to undergo fertilization. (A–I) Confocal micrographs of *ACT11:MSI1:GFP* ovules receiving pollen tubes expressing a sperm-specific *HTR10:HTR10:RFP*, 16 h after pollination. Scale bars=20 μm. (A–C) Wild-type *HTR10:HTR10:RFP* sperm cells are visible in ovules with a diffuse signal (A) (arrows) that overlaps with the nuclear GFP signal (B) from the egg and central cell indicating the successful fusion of gametes and formation of the zygote nucleus (zn) and endosperm nucleus (en). The synergid nucleus of the receptive synergid has degenerated (dsn) and the non-receptive synergid nucleus is still intact (sn), indicating that pollen tube reception has taken place normally. (C) Overlapping RFP and GFP signal. (D–I) Ovules receiving *chx17chx18chx19*, *HTR10:HTR10:RFP* sperm cells frequently lack positive signal from the *HTR10:HTR10:RFP* reporter. (D) Sperm cell signal of *HTR10:HTR10:RFP* is visible in pollen grains that have detached from the stigma into the media (pollen grains, top left), yet not inside ovules. (E) Ovules show evidence of pollen tube reception and synergid degeneration by the degeneration of one or both synergid nuclei (dsn). (F) Overlapping GFP and RFP signals indicate a loss of visible RFP signal, yet the egg (ecn) and central cell nuclear (ccn) GFP signal remains strong. (G) Overlapping RFP and GFP signal from an ovule with two sperm cells (sc) approaching the micropyle, indicating normal pollen tube arrival at the ovule prior to reception. Ovules with one (H) or two (I) degenerated synergid nuclear GFP signals (dsn) and one visible *HTR10:HTR10:RFP*-positive sperm cell that has failed to undergo fusion with either the egg or the central cell. (J) Quantification of the *HTR10:HTR10:RFP* sperm signal in all ovules receiving wild-type or *chx17,18,19* pollen tubes. (K) Quantification of nuclear *ACT11:MSI1:GFP* signal in synergids in ovules from (J), 0 SN, zero intact synergid nuclei; 1 SN, one intact synergid nucleus; 2 SN, two intact synergid nuclei. The total number of ovules observed for each category is written above each stacked column, and the number of ovules in each synergid status category is written on each stacked column unit.

It is clear that ovules are targeted by *chx* triple mutant pollen tubes (Fig. 4), yet fail to develop into seeds (Fig. 5). However, given the expression pattern (Fig. 5G) of *CHX17*, *CHX18*, and *CHX19*, this could be due to a defect in interactions between mutant pollen tubes and female gametophytes, or to failed interactions between mutant sperm and female gametes. To resolve the spatial and temporal relationship of pollen tube interactions with the embryo sac and the success of sperm fusion with the female gametes, we used an assay that simultaneously monitors synergid degeneration and the status of sperm cells (Leydon *et al.*, 2015). After a pollen tube enters and contacts the ovule, one of two synergid cells degenerates. Plants carrying *ACT11:MSI1:GFP* express nuclear GFP in all cells, but the synergid cells express it most strongly (Fig. 6B). As the receptive synergid degenerates, the nuclear GFP deteriorates and loss of the nuclear integrity is especially clear when the GFP signal diffuses throughout the synergid cytoplasm (Fig. 6B). After wild-type *ACT11:MSI1:GFP* pistils were pollinated with wild-type pollen carrying *HTR10:HTR10:RFP*, which labels sperm nuclei (Ingouff *et al.*, 2007), 81% of ovules showed either one or no intact synergid nuclei, and diffuse HTR10:RFP signal overlapping the egg cell nucleus and central cell nucleus, indicative of successful pollen tube reception and double fertilization (Fig. 6A–C, J–K). The remaining 19% of ovules had both synergid nuclei intact, and were probably untargeted by pollen tubes (Fig. 6J, K). In contrast, when wild-type *ACT11:MSI1:GFP* pistils were pollinated by the *chx* triple mutant, 95.3% of ovules had no visible sperm cells, though 334 out of 443 ovules showed that at least one synergid had undergone degeneration (Fig. 6D–F, J, K). The degeneration of the synergid cell nuclei indicates that mutant pollen tube had reached the synergid cell, consistent with the observation of sperm cells localized to the micropylar region of the ovule (Fig. 6G). Unfused sperm cells were observed in 3.2% (or 14) of the ovules (Fig. 6H, I); these ovules showed synergid degeneration with no sign of plasmogamy; in many cases, the sperm appeared to be deposited incorrectly at the micropylar end of the synergid, rather than at the interface between the egg and the central cell where gamete fusion takes place (Hamamura *et al.*, 2011). Only 1.6% (seven) ovules showed successful double fertilization and had either one or no intact synergids (Fig. 6J, K). Thus the frequency of double fertilization was extremely low in *chx* triple mutant pollen, though the frequency of synergid degeneration is high. Thus *chx* triple mutant pollen tubes can target ovules and undergo normal pollen tube reception as evidenced by synergid degeneration. Therefore, reduced male fertility of *chx17chx18chx19* triple mutants is due to partial failure in pollen discharge, sperm positioning, impaired sperm–egg interactions, sperm degeneration during pollen tube growth, or a combination of these defects.

### Delayed embryo development of self-pollinated triple mutants

**Fig. 7.**
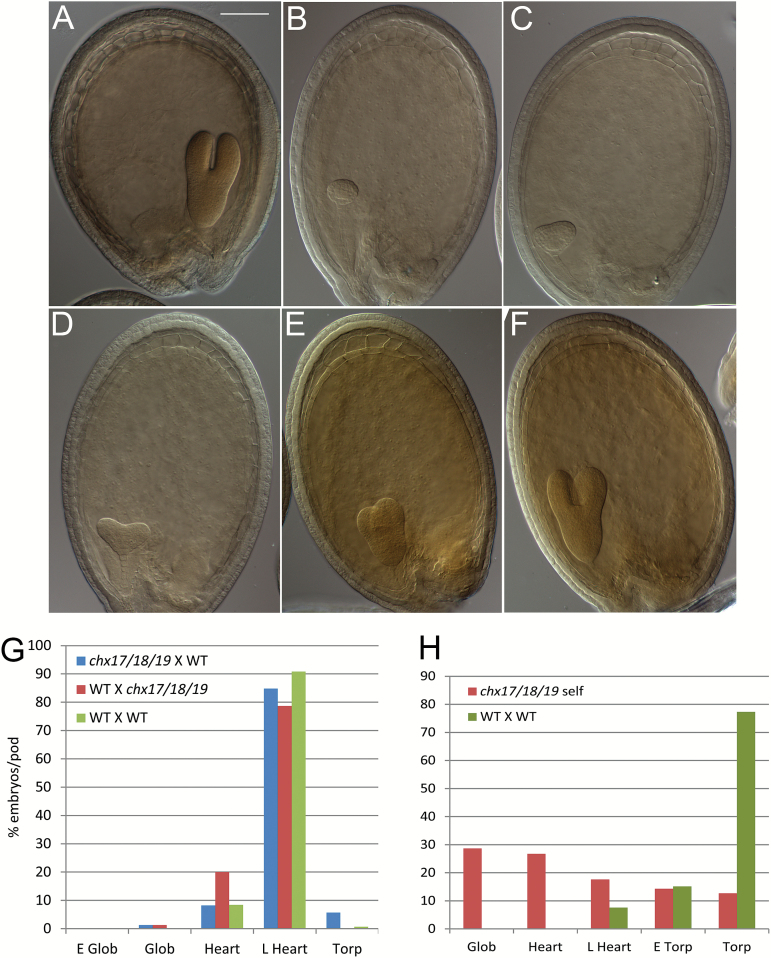
The self-pollinated *chx17chx18chx19* mutant also show delayed embryo development when fertilization is successful. Six days after pollination, pods were fixed and cleared with chloral hydrate. (A) Wild-type (WT) embryos are mostly at the torpedo stage. (B–F) Mutant ovules at various developmental stages from a single pod: (B) globular, (C) early heart, (D) late heart, (E) early torpedo, and (F) torpedo. Scale bars=100 μm. (G) The embryo developed to a similar stage when a wild-type pistil received *chx17/18/19* mutant pollen or vice versa. The percentage of embryo stages per pod from (i) WT×WT cross; (ii)WT pistil×mutant pollen; or (iii) mutant pistil×WT pollen. (H) Self-pollinated triple mutants showed delayed embryo development. WT or triple *chx* mutants were self-pollinated and pods were examined by DIC microscopy. Results are the average of 10 pods per treatment.

Most embryos within a wild-type silique develop at similar rates, so they reach the late heart stage by 5 DAP (Fig. 7A). Similarly, most of the embryos (80–90%) produced from a wild-type pistil pollinated with triple mutant pollen developed to the late heart stage by 4–5 DAP (Fig. 7G). However, when fertilization was successful in self-pollinated triple mutants, embryos within a silique showed a range of developmental stages (Fig. 7B–E). When some embryos had reached the late torpedo stage, others were at the globular or early to late heart stages (Fig. 7H). Thus, homozygous triple mutant embryos showed delayed development. From six pods, we estimated that ~55% of the embryos had not reached the heart stage, when 30% were at the early to torpedo stage. The fate of embryos with severely delayed development was not followed so it is unclear whether development would abort prematurely, or progress to full maturity. These results suggest that CHX functions are also critical for post-fertilization development of the young sporophyte. As a wild-type pistil pollinated with triple mutant pollen produced embryos that developed similarly to the wild type, our results suggest that at least one copy of *CHX17*, *CHX18*, or *CHX19* is required for synchronous development of mutant embryos.

## Discussion

We take advantage of a *chx17chx18chx19* mutant to dissect the basis of reduced seed set and shed light on the specific reproductive phase(s) affecting male fertility. Reduced male fertility is attributed to multiple steps, including germination, tube growth, pollen discharge, and fertilization defect in sperm. Our studies highlight the importance of endomembrane transporters involved in pH and cation homeostasis. Combined with previous functional studies, these results suggest that CHX transporters influence reproductive development through membrane trafficking by possibly remodeling cell walls and the PM.

### Male transmission defect and failed fertilization

Based on reciprocal crosses and the low transmission of the mutant *chx18* or *chx19* allele (3–5% instead of 50%) by the male gametophyte to the F_1_ progeny, we concluded that a male gametophyte with loss of function in *CHX17*, *CHX18*, and *CHX19* genes was severely impaired. In contrast, transmission of the mutant *chx18* or *chx19* allele by the female gametophyte was 25–30% instead of 50%, thus a triple mutant female gametophyte was less severely compromised.

The basis of the male gametophyte defect was apparently not due to development of the microspore into the mature pollen based on light microscopy. The male gametophyte begins after meiosis of a pollen mother cell in the anther, forming four haploid microspores. Each microspore divides to produce a bicellular pollen containing a vegetative cell and a generative cell. The generative cell then undergoes mitosis to produce two sperms cells, thus giving rise to a tricellular mature pollen. DAPI staining showed that 95% of triple mutant grains contained three nuclei, similar to those of wild-type grains, indicating that male gametogenesis was largely unaffected by loss of CHX17, CHX18, and CHX19 activity. Ultrastructural studies confirmed that the mutant developed like the wild type (not shown); however, SEM revealed a disorganized wall in mutant pollen.

Mutant pollen had a ‘spongy’ wall instead of a reticulate pattern, though that defect alone is unlikely to reduce male fertility (Dobritsa *et al.*, 2011). The outer wall of pollen grains protects against dessication, and enhances adhesion and hydration on the stigma before pollen germination. Germination of *chx*1*7chx18chx19* pollen was reduced and tube lengths were shorter *in vitro*; yet the quantity of pollen *in vivo* ensured that sufficient grains attached to the stigma and many tubes grew into the ovary. Thus pollen functions in the early phases of reproduction were not severely hampered. Aniline blue staining demonstrated that mutant pollen tubes had targeted and entered many ovules, indicating that loss of CHX17, CHX18, and CHX19 function did not affect the ability of tubes to sense cues secreted by the female gametophyte and to target the embryo sac (Higashiyama and Takeuchi, 2015).

Pollen tubes were observed in both developing and undeveloped seeds, suggesting that the pollen tube had entered the micropyle in both cases. About 39–65% of undeveloped ovules in each pod had received a pollen tube, indicating that fertilization was unsuccessful. Moreover, mutant pollen tube reception was verified by the degeneration of one or both synergid cells seen in 80% ovules of a wild-type pistil (Fig. 6K). In wild-type pollen, both sperm cells are released simultaneously at tube rupture after synergid cell reception (Hamamura *et al.*, 2011). One sperm fuses with the egg cell to form a zygote that develops into the embryo. The second sperm nucleus unites with the central cell nucleus and undergoes subsequent nuclear division and cellularization to produce the endosperm. Direct analysis of *chx* triple mutant sperm cells in female gametophytes suggests that many pollen tubes may fail to deliver sperm. The vast majority of ovules pollinated with triple mutant pollen had no sperm cells visible based on RFP fluorescence (Fig. 6K). Failure to express markers of sperm identity such as *HTR10:HTR10:RFP* (Fig. 6) may indicate that mutant sperm cells are compromised. The lack of visible sperm signal could account for the apparent low frequency of successful fertilization estimated from the imaging results (Fig. 6) compared with 27–45% of developing seeds per pod in pistils receiving *chx* mutant pollen *in vivo* (Fig. 4).

Undeveloped ovules that received a pollen tube suggest that either tube rupture was defective, or released sperm cells fail to complete fertilization with the female gametes, or both. Single fertilization events were observed, indicating that defective tube rupture is not the major cause. Some ovules develop only a globular embryo with no detectable endosperm tissue, and other ovules enlarge in size due to proliferation of the endosperm nuclei, with no visible embryo. Either of these examples will lead to abortion and failed seed development, as embryogenesis and endosperm development are co-ordinated to produce successful seed development (Berger, 2003). This idea is verified by the presence of unfused sperm cell in ovules pollinated by *chx17chx18chx19* mutant pollen (Fig. 6H, I, K). Single fertilization events suggest that one of the two sperm failed to complete fertilization. Thus loss of *CHX17*,*CHX18*, and*CHX19* genes compromised sperm function in half to two-thirds of the male gametophytes, leading to failed double fertilization.

The ability of any one of three *CHX* genes to rescue male fertility is unexpected as they are differentially expressed in space and time. *CHX17* transcript detected in microspore and bicellular pollen (Honys and Twell, 2004) was confirmed by CHX17p::GUS (β-glucuronidase) staining of anthers in stage 9–10 flowers (Bock *et al.*, 2006). *CHX19* transcript is detected in developing pollen grains and tubes (Fig. 5G), where the protein was localized to the PM (Supplementary Fig. S6). *CHX18* transcript was detected in sperm cells (Fig. 5G) isolated by fluorescence-activated cell sorting (Borges *et al.*, 2008), whereas *CHX19* expression is low or ‘absent’. In contrast, *CHX18* transcript was low or not detectable in mature pollen (Honys and Twell, 2004) or in the tube (Qin *et al.*, 2009), suggesting that *CHX18* transcript in sperm cells is not contaminated by mRNA from the vegetative cell. *HAP2/GCS1* or *GEX2* are experimentally demonstrated to be sperm-expressed genes (Mori *et al.*, 2006, 2014; von Besser *et al.*, 2006), whereas Feronia is not expressed in pollen and is expressed in synergid cells of the female gametophyte (Huck *et al.*, 2003; Rotman *et al.*, 2003; Escobar-Restrepo *et al.*, 2007). The transcriptome results of reproductive tissues have been verified by these studies (Fig. 5G), indicating that *AtCHX18* and *AtCHX17* are expressed in sperm cells. At least one of three CHXs is expressed at every stage of male gametophyte development. Apparently one copy of *CHX19* is sufficient to restore male fertility of a *chx17chx18* double mutant. Thus plants have mechanisms to compensate when one or more homologous genes are rendered non-functional.

Yet some *chx17chx18chx19* mutants completed double fertilization successfully. This partial success in fertilization by triple mutant sperm could be due to compensation by other sperm-expressed cation/H^+^ exchangers, such as AtCHX20 (see Fig. 5G) or AtNHX2. AtCHX20 also mediates cation and pH homeostasis in yeast (Padmanaban *et al.*, 2007), though it differs from CHX17, in endomembrane localization and inability to sort cargo, such as aminoglycoside HygB, in yeast (Chanroj *et al.*, 2011).

### The female gametophyte and post-fertilization events contribute to seed development

The basis for transmission of the *CHX18*^*+*^ allele via the female gametophyte (Table 2) awaits further investigation. Both *CHX17* and *CHX18* transcripts were detected in the central cell (Schmid *et al.*, 2012) (Supplemenatry Fig. S7), and possibly in the synergid and egg cell (Wuest *et al.*, 2010; S. Sprunck, personal communication).

We showed that one functional *CHX17*, *CHX18*, or *CHX19* is also critical for post-fertilization development, as early embryo development contained in one pod was synchronous in a heterozygous *chx17*^*+/–*^*chx18*^*+/–*^*chx19*^*+/–*^ sporophyte but was delayed in a homozygous *chx17*^*–/–*^*chx18*^*–/–*^*chx19*^*–/–*^ mutant (Fig. 7G, H). We suggest that after fertilization, early embryo development depends on a functional CHX17 or CHX18 in the embryo, endosperm, or both. Several observations support this idea. First, CHX17p::GFP expression is strong at the anterior end of the developing seed (Chanroj *et al.*, 2013) consistent with *CHX17* expression at the micropylar endosperm of the early embryo (Belmonte *et al.*, 2013). Secondly, *CHX18* is highly expressed in the micropylar and the peripheral endosperm at the pre-globular and globular embryo stage according to the transcriptome of Arabidopsis seed development (Supplementary Fig. S8) (Belmonte *et al.*, 2013). Thirdly, RNA sequencing revealed *CHX17* and *CHX18* transcripts in a laser-dissected central cell (Supplementary Fig. S7) (Schmid *et al.*, 2012). Union of a sperm cell with the central cell nucleus and subsequent proliferation produces a micropylar endosperm that surrounds the embryo. The role of the micropylar endosperm is unclear. A recent study demonstrated that cysteine-rich peptides, ESF1 (Embryo Surrounding Factor) accumulated in the central cell before fertilization and in the micropylar endosperm after fertilization (Costa *et al.*, 2014). Furthermore, purified ESF has a role in early development of the embryo, possibly through suspensor elongation and auxin distribution. These results suggest that the endosperm surrounding the embryo plays a critical role in producing peptide cues to facilitate development of the suspensor and pre-globular embryo. One model is that CHX17 and CHX18 influence peptide sorting and secretion from the micropylar endosperm to the target cells.

### Model: CHX17, CHX18, and CHX19 activities affect PM and wall remodeling

How would loss of CHX17, CHX18, or CHX19 produce the various defects we observed? The loss in exine patterning of *chx17chx18chx19* mutant grains may be indirectly related to decreased male fertility, yet it may provide a clue to compromised wall formation. There are two schools of thought regarding outer wall formation: (i) primexine (precursor of exine) and exine seen on microspores depend on materials synthesized from tapetum (or sporophyte); and (ii) primexine and exine are formed by the combined activities of the microspore and tapetum (Dobritsa *et al.*, 2011). Ultrastructural studies had suggested that primexine originated from the PM of the microspore. For instance, the *dex1* mutant is male-sterile, and a tetrad of *dex1* microspores surrounded by callose wall shows defects in early primexine formation and later collapsed pollen grains (Paxson-Sowders *et al.*, 2001). *DEX1* is expressed in microspores and encodes a predicted integral protein. Curiously, the sterility defect followed Mendelian inheritance, suggesting control by the maternal sporophyte (Paxson-Sowders *et al.*, 2001). Our genetic studies show that loss of male fertility in *chx17chx18chx19* mutants is largely a male gametophyte defect and is accompanied by exine deformation. While we cannot eliminate CHX function in tapetal or pollen mother cells, our SEM results (not shown) support the hypothesis that primexine synthesis is initiated from microspores. It is likely that microspores within a tetrad produce a scaffold or template upon which exine materials attach and assemble (Ariizumi and Toriyama, 2011). Thus, any defects in the scaffold could directly affect subsequent exine patterning. Alternatively, a defect in intine (or inner wall) that is formed later by bicellular pollen between the PM and the outer wall might perturb exine patterning. These ideas warrant reconsideration.

How could three CHX transporters with nearly identical activities perturb male fertility? To date, our results are consistent with the hypothesis that *chx* mutants have perturbed membrane trafficking that remodels the PM and the cell wall (Kim and Brandizzi, 2016), and those changes lead to impaired fertility. First, *CHX17* as well as *CHX18* or *CHX19* confer tolerance to alkaline pH in a yeast mutant sensitive to growth at pH 7.5, suggesting a role in pH homeostasis. Secondly, *CHX17* supports growth of K^+^ uptake mutants in yeast and *E. coli*, suggesting a role in K^+^ transport and K^+^ homeostasis. Thirdly, CHX17 could reduce the secretion of vacuolar carboxypeptidase Y in yeast, indicating that it has a role in proper sorting of cargo (Chanroj *et al.*, 2011). As CHX17 has been localized to the PVC and the PM in plant cells (Chanroj *et al.*, 2013), the results infer that proper regulation of the endomembrane pH and cation level plays a role in protein and cargo sorting (Chanroj *et al.*, 2011). This idea is demonstrated convincingly in a *det3* H^+^-pumping V-ATPase mutant that showed increased pH in the *trans*-Golgi network (TGN), and altered secretion and recycling of cellulose synthase to the PM (Luo *et al.*, 2015).

Our results show that *chx17chx18chx19* mutant pollen is compromised at multiple phases in reproduction. As many ovules received a pollen tube, we suggest that failed fertilization is mainly due to reduced tube rupture or impaired sperm activity, or both. Loss of function of PM-localized CHX19 could affect tube growth and rupture perhaps through ANX1/ANX2 receptor-like kinases (Boisson-Dernier *et al.*, 2009). Loss of *CHX18* and *CHX17* function in sperm cells could impair male gamete activity. CHX17 was shown to alter sorting of protein cargo in yeast (Chanroj *et al.*, 2011), thus plasma membrane proteins (e.g. receptors) or secreted factors of the pollen tube or sperm might be misguided in the triple mutant.

HAP2 is a sperm cell-expressed PM protein that is crucial for fertilization (Mori *et al.*, 2006; von Besser *et al.*, 2006). HAP2 is a transmembrane protein that is sorted to the PM from the ER after activation by EC1, an egg cell-secreted peptide that promotes fertilization (Sprunck *et al.*, 2012). A homolog in *Chlamydomonas* is needed for cell–cell fusion, suggesting that the plant *HAP2/GCS1* might act as a gamete fusogen (Liu *et al.*, 2008). *GEX2* encodes a PM-localized protein in sperm cells. Mutant sperm cells failed to attach to the egg membrane, indicating that GEX2 is required for gamete–gamete attachment, a prerequisite for successful fusion (Mori *et al.*, 2014). Perhaps sperm CHX18 influences the sorting and delivery of proteins, such as GEX2 or HAP2 (GCS1), to promote gamete attachment and fusion.

Our findings support a model in which three related cation/H^+^ exchangers affect a subset of endomembranes in developing pollen, pollen tube, sperm, and central cells. Pollen development, tube sensing, or wall properties might be sufficiently compromised to reduce sperm release, sperm function, or both in *chx17chx18chx19* mutants. Our study highlights the critical roles of cation/H^+^ exchangers in membrane trafficking, cargo sorting, and wall remodeling for successful fertilization.

## Supplementary data

Supplementary data are available at *JXB* online.

Table S1. Single T-DNA insertion mutants used in this study.

Table S2. Gene-specific primers and T-DNA border primers used for genotyping plants.

Fig. S1. Flower development of the *chx17/18/19* mutant and the wild type is similar.

Fig. S2. Pod and seed development at 0–9 days after pollination.

Fig. S3. Pollen wall architecture is partially disorganized in *chx17/18* double mutants but not in the single *chx17* mutant.

Fig. S4. Time-course of ovule development in wild-type (WT) pistils receiving WT or *chx17/18/19* mutant pollen.

Fig. S5. Pollen tubes of the *chx17/18/19* mutant entered ovules that remain undeveloped.

Fig. S6. CHX19–RFP was localized to the PM along the flanks of the pollen tube.

Fig. S7. RNA sequencing revealed expression of CHX17 and CHX18 in a laser-dissected central cell.

Fig. S8. Expression of *CHX17* and *CHX18* in the micropylar and peripheral endosperm during early seed development.

## Author contributions

HS conceived the original research plans; HS, AYC, MAJ supervised the experiments; SP, DC, and RS performed most experiments, KL, TKM, ARL, and YZ performed other experiments; SC generated the mutants; SP, DC, ARL, and HS designed the experiments and analyzed the data; HS wrote the article with contributions of all the authors; and AYC and MAJ complemented the writing.

## Supplementary Material

supplementary_tables_S1_S2_figures_S1_S8
