# Supplementary material for: Transporters involved in pH and K+ homeostasis affect pollen wall formation, male fertility, and embryo development
Source: J Exp Bot. 2017 Feb 23;68(12):3165–78. doi: 10.1093/jxb/erw483 (PMC5853877; doi:10.1093/jxb/erw483)
Supplement: supplementary_tables_S1_S2_figures_S1_S8 [file erw483_suppl_supplementary_tables_s1_s2_figures_s1_s8.pdf]

Table S1. Single T-DNA insertion mutants used in this study.
All mutant lines were generated in the Columbia-0 ecotype.

**Mutant**	**SALK Line #**	**T-DNA Insertion site (after base)**
chx16-10a	SALK_138136	767
chx17-4	SALK_033417	996
chx18-1	SALK_001563	2070
chx19-2	SALK_100047	619

Table S2. Gene-specific primers and T-DNA border primers used for genotyping plants.

**Name**	**Sequence**	**Product detected**
chx17-4TLP	AACTTTGCAGTAAAGGGAAACTAGGACTG	genomic CHX17
chx17-4TRP	TGAGTTTTATATTGTGGTTTTAGGGTGGA	genomic CHX17<br>*chx17-4* T-DNA (b1.3)
chx18-1TLP	ATTCTACCAACCAAAAACAAATTGCTC	genomic CHX18<br>*chx18-1* T-DNA (b1)
chx18-1TRP	AGAAGAGCGAAGAAAGAAGGAGAATACAA	genomic CHX18
chx19-2LP	TGTAAGTAGATCTCCACTAGGATGGCAAG	genomic CHX19
chx19-2RP_n2	GCGCGTGTATCCCAATAGTGTCTGTC	genomic CHX19<br>*chx19-2* T-DNA (b1)
LB-b1.3	ATTTTGCCGATTTCGGAAC	*chx17-4* T-DNA
LB-b1	GCGTGGACCGCTTGCTGCAAC T	*chx18-1* and *chx19-2* T-DNA

II. RESULTS

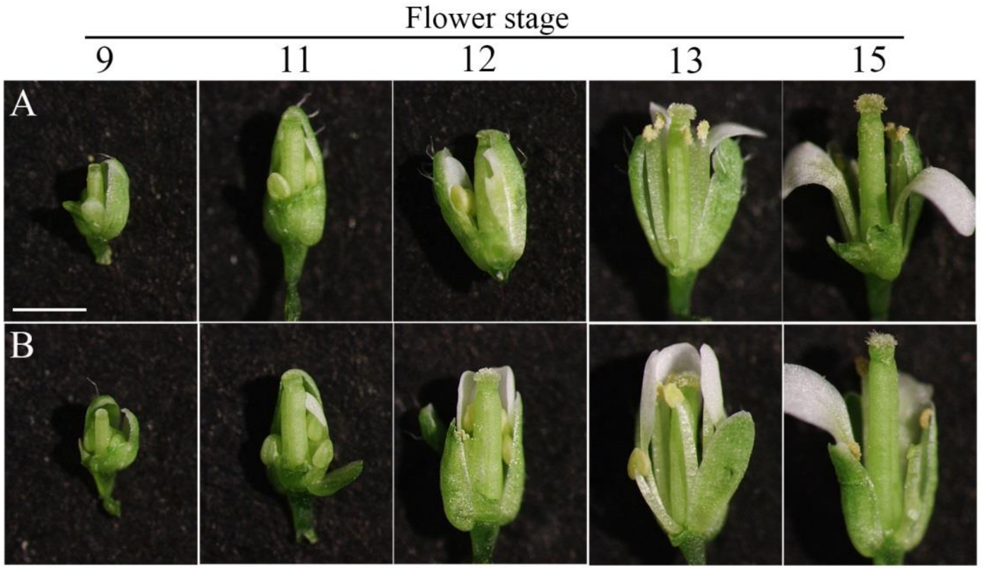

Fig. S1. Flower development of *chx17/18/19* mutant and WT is similar. Flowers from stages 9, 11, 12, 13, and 15 were collected from the primary bolts of WT **A)** and *chx17/18/19* **B)** plants. Sepals and petals were removed from each flower to visualize stamens and pistil. Scale bar = 1 mm.

Fig. S2. Pod and seed development at 0 to 9 days after pollination.

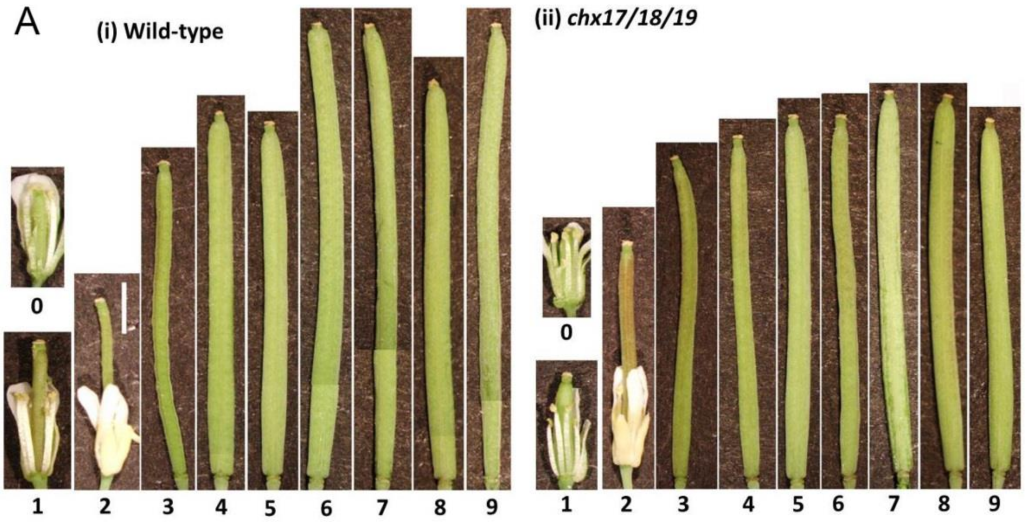

A) Pod elongated rapidly 2-3 days after pollination (DAP). Seed pods were removed from wild-type (i) and *chx17/18/19* (ii) plants at 0 to 9 DAP. Wild-type pods at 5 to 9 DAP is longer than *chx17/18/19* mutant pods. Scale bar = 2 mm. Long pods are shown as composite images taken at the same magnification.

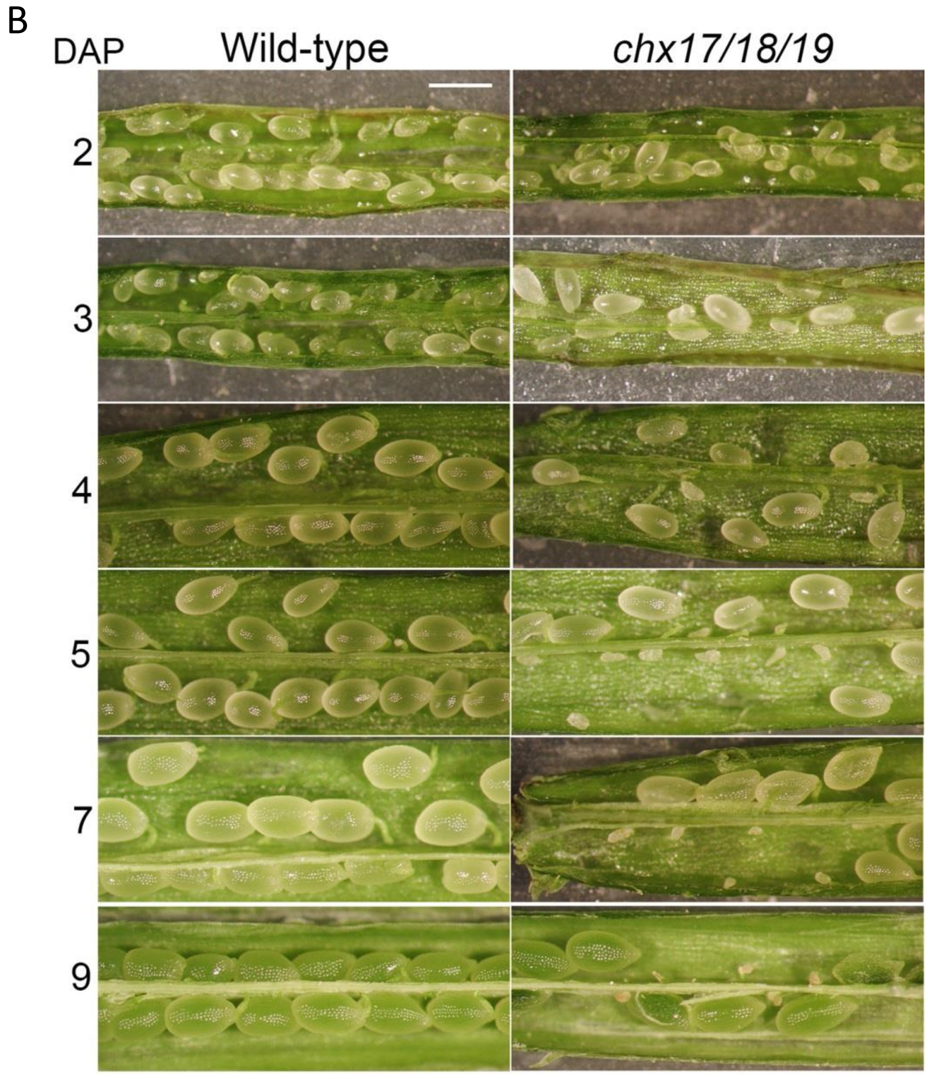

B) Opened pods of WT and *chx17/18/19* at 2 to 9 DAP. WT pods are filled with developing seeds. Mutant pods show gaps where ovules failed to increase in size by 3-4 DAP. After 5-6 DAP, undeveloped ovules begin to decrease in size and shriveled by 8-9 DAP. Four seed pods were examined for each stage. Scale bar = 0.5 mm.

Fig. S3.

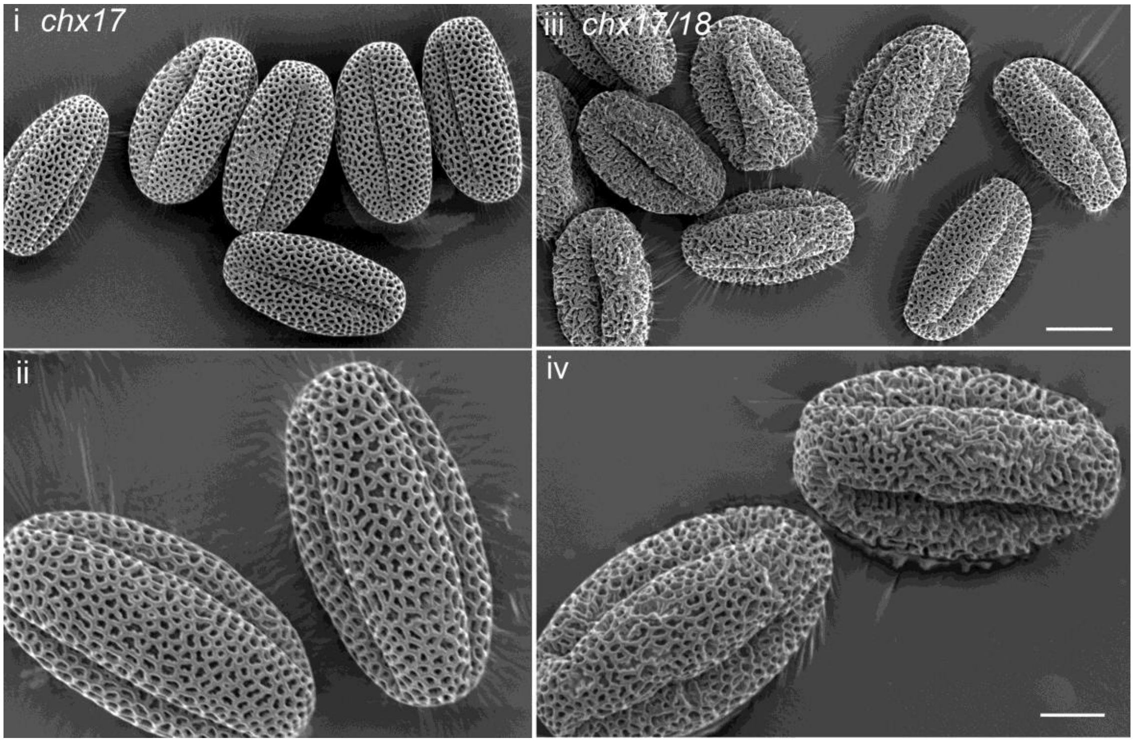

Fig. S3. Pollen wall architecture is partially disorganized in *chx17chx18* double mutants but not in single *chx17* mutant. Scanning electron micrographs of mature pollen grains from single *chx17* mutants (I, ii) and from double *chx17/18* mutants (iii, iv). Half of the double mutant grains (52%) had disorganized walls, 16% were intermediate, and 31% looked nearly like wild-type. Scale bar is 10 μm (i, iii) or 5 μm (ii, iv).

Fig. S4

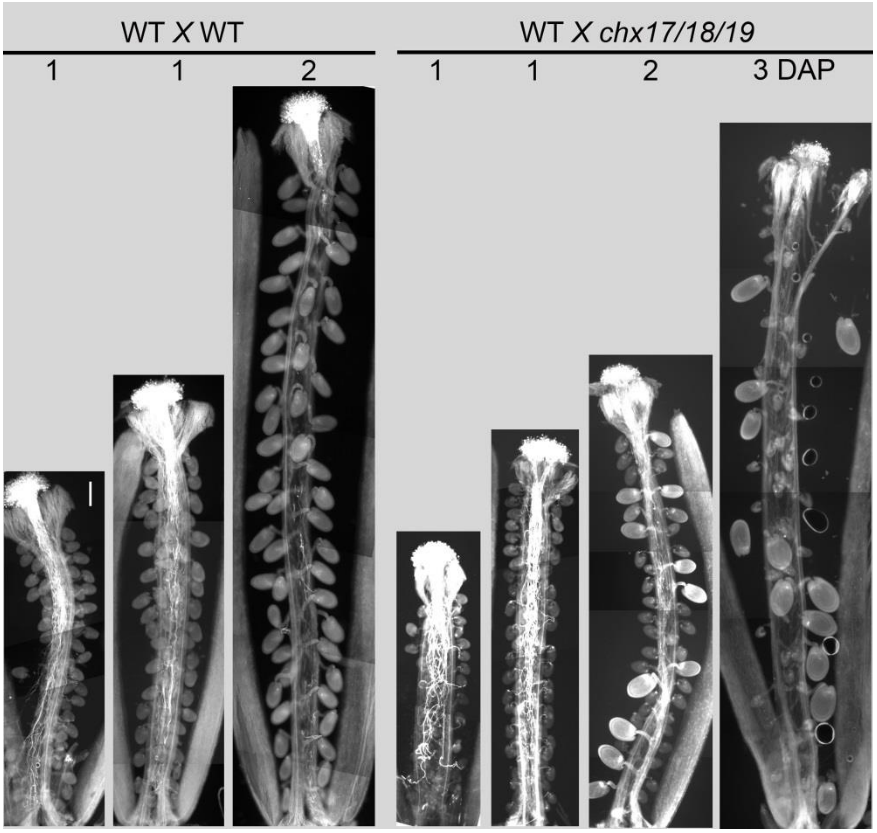

Fig. S4. Time-course of ovule development in wild-type (WT) pistils receiving WT or *chx17/18/19* mutant pollen. WT pistils were pollinated with WT (left) or *chx17/18/19* mutant pollen (right). Pods were fixed at 1 to 3 DAP. Siliques were stained with aniline blue, split open and observed by fluorescent microscopy. Several images were taken at the lowest magnification (10x objective) and put together to show the entire carpel.

Fig. S5

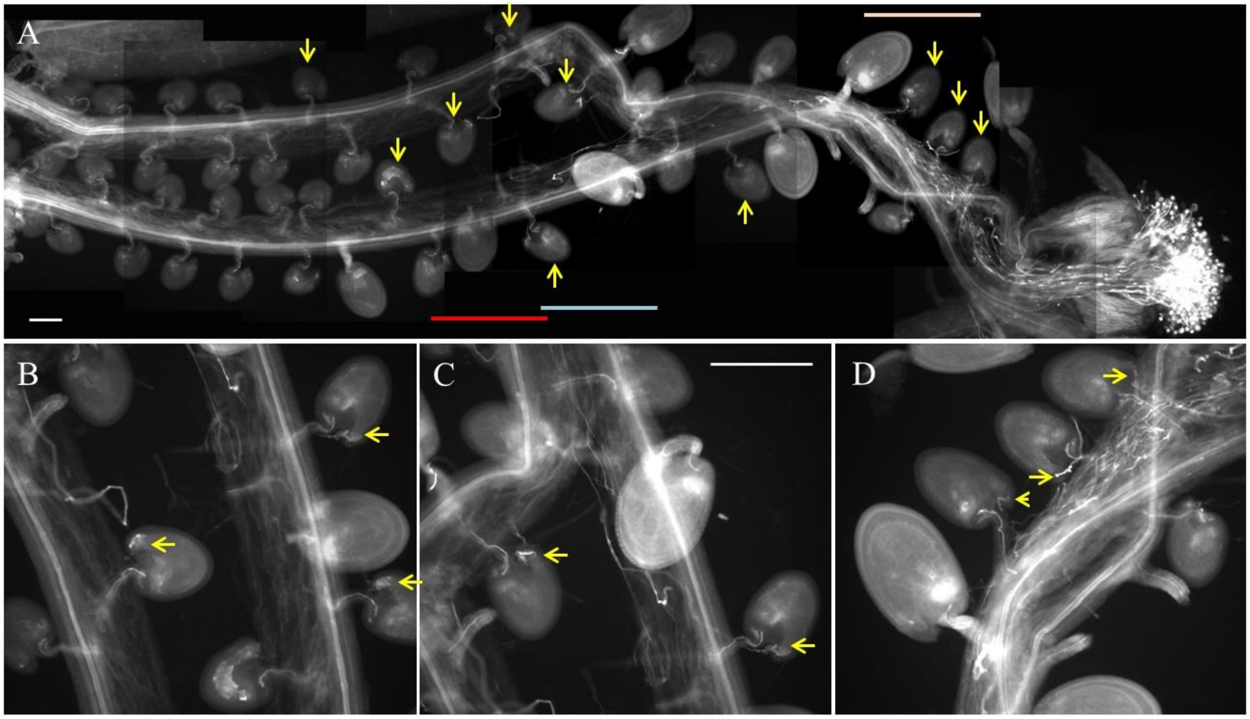

Fig. S5. Pollen tubes of *chx17/18/19* mutant entered ovules that remain undeveloped. A WT pistil was pollinated with *chx17/18/19* pollen. Pod was examined at 2 DAP. **A)** Yellow arrows point to ovules containing a pollen tube (PT). **B-D)** Examples of magnified images show PTs visible inside ovules that remain undeveloped. PTs in large developing seeds are often no longer visible. The red line in panel A represents the magnified image in panel B. The blue and peach lines in A represent magnified images in C and D, respectively. Scale bar (white) = 100 µm. Panel A is composed of images taken at the lowest magnification (10x objective) and put together to show the entire developing pod. B-D panels are single images taken at 20x objective.

Fig. S6

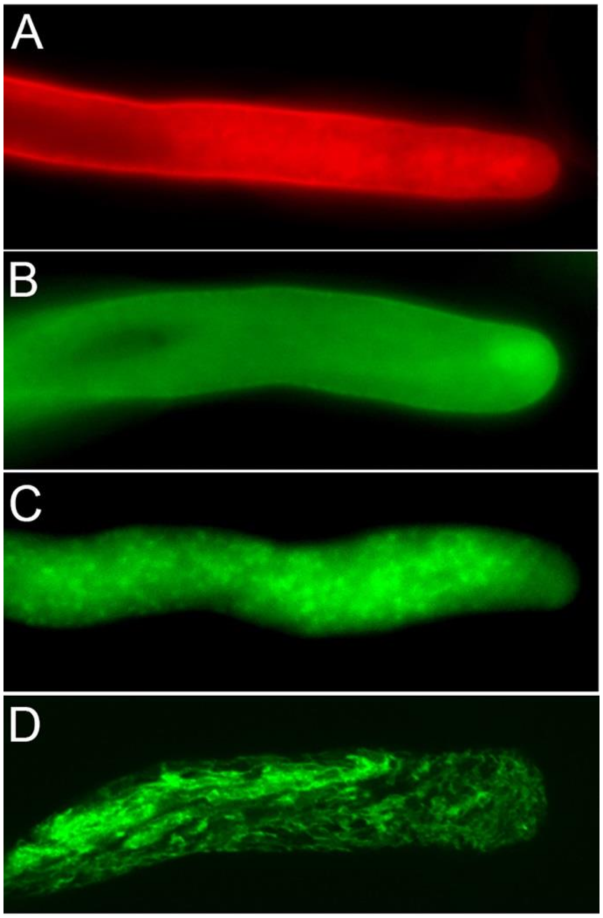

Fig. S6. CHX19-RFP was localized to the PM along the flanks of the pollen tube. Tobacco pollen was bombarded with various constructs encoding CHX19 or a marker fused with a fluorescence protein. Grains were germinated and tubes were observed by microscopy. A) CHX19-RFP, B) GFP-PRK1, PM marker; C) GFP-Rab2, Golgi marker; and D) GFP-HDEL, ER marker. (SP with Yanjiao Zou)

Fig. S7.

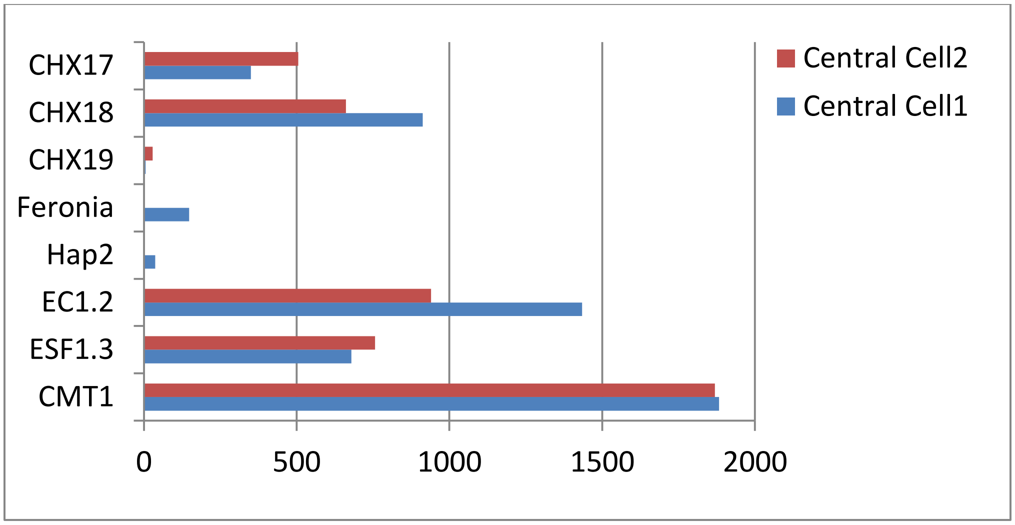

ATG #	Protein	Central Cell1	Central Cell2
AT1g80740	CMT1	1883	1869
At1g10717	ESF1.3	679	756
AT2g21740	EC1.2	1434	939
AT4g11720	Hap2	37	0
AT3g51550	Feronia	148	0
AT3g17630	CHX19	6	29
AT5g41610	CHX18	913	661
AT4g23700	CHX17	350	505

Fig. S7. RNA-sequencing revealed expression of CHX17 and CHX18 in laser-dissected central cell. Top figure is based on results of Schmid et al. (2012) shown in Table below. ESF1.2 and ESF1.3 are Embryo Surrounding Factors which are cysteine-rich peptides demonstrated to be in the central cell in unfertilized embryo sac and in the micropylar endosperm after fertilization (Costa L et al. 2014). CMT1 is a chromomethylase involved in epigenetic functions, and its transcripts are enriched in the central cell (Schmid et al., 2012). Feronia is a synergid cell expressed Receptor-Like Kinase. Hap2 is a sperm-expressed membrane protein. EC1.2 is a DEFL protein expressed in the egg cell (Sprunk et al 2012), its detection in central cell needs to be verified.

Fig. S8.

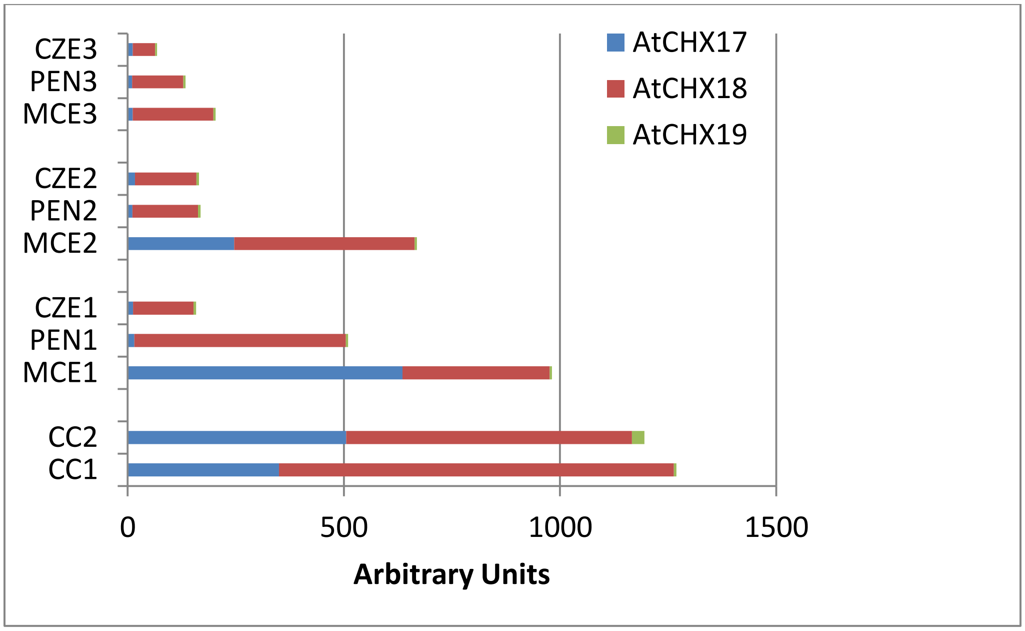

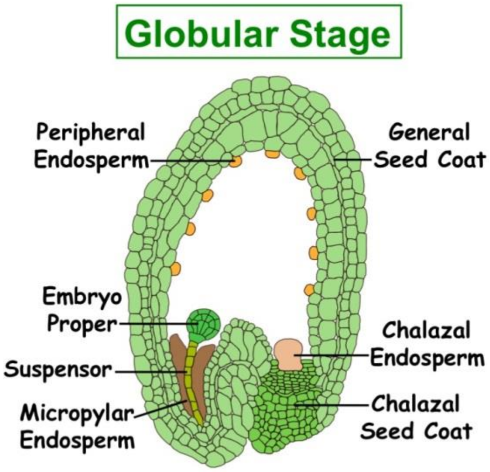

Fig. S8. ***CHX17* and *CHX18* expression in the micropylar and peripheral endosperm of early seed development**. Expression of 3 CHX genes shown as color-coded bar is based on the central cell (CC1 & CC2) taken from two RNA-sequencing experiments of Schmid et al. (2012). Relative expression in the micropylar endosperm (MCE), peripheral endosperm (PEN) and chalazal endosperm (CZE) are taken from ATH1 microarray data of Belmonte et al. (2013). Numbers following MCE, PEN or CZE refer to laser-dissected tissues from preglobular (1), globular (2), and mature green (3) embryos. Section of the seed at the Globular Stage is taken from ‘seedgenenetwork.net’.
